# Supplementary material for: Identification of Freezing-Responsive microRNAs and Their Targets in Chinese Jujube by Small RNA and Degradome Sequencing
Source: Int J Mol Sci. 2026 Sep 4;27(17):7912. doi: 10.3390/ijms27177912 (PMC13566890; doi:10.3390/ijms27177912)
Supplement: Supplementary file 1 [file ijms-27-07912-s001.zip › Supplementary Material S2/novel_miR_58.pdf]

Provisional ID	:	novel_miR_58
Score total	:	24.1
Score for star read(s)	:	3.9
Score for read counts	:	21.5
Score for mfe	:	0.9
Score for randfold	:	-2.2
Score for cons. seed	:	
Total read count	:	54
Mature read count	:	50
Loop read count	:	3
Star read count	:	1

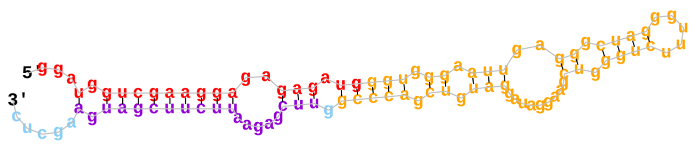

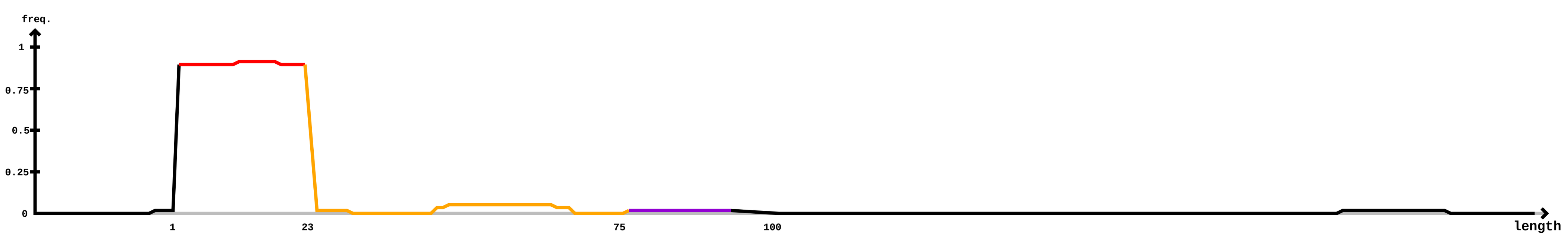

Mature Star

```
5'- caugagcuugguggguggguaaggauggucgaaggagagagaugggugggaauugagggcuaggguuucugggucgaaggauaggaugucgacccgguucgagaauucuucgaugaagcucccgacggcgauucgguggugggcgcguucgugccggugaggaggaggacaaggccauggcguauaaaggauaaggggauucauaagucaccgaauuaucggaucugagaugagauuccugcgucgaauuu -3' obs
    caugagcuugguggguggguaaggauggucgaaggagagagaugggugggaauugagggcuaggguuucugggucgaaggauaggaugucgacccgguucgagaauucuucgaugaagcucccgacggcgauucgguggugggcgcguucgugccggugaggaggaggacaaggccauggcguauaaaggauaaggggauucauaagucaccgaauuaucggaucugagaugagauuccugcgucgaauuu     exp
    ..((((.(((.((..((((..((..(.(((((((((..(((.(((((.((.(((..((.((((.....)))).))........))).)).))))).)))....))))))))).)..)))))).)).)))))))((.(((((..((((...((.....))....))))...))))).))........(((..((((((((((...(((((((....))))...))).)))).))))))..)))......  reads  mm  sample
    ..................guaaggauggucgaaggagag..................................................................................................................................................................................................................  1      0   LT-1
    ......................ggauggucgaaggagagagaug.............................................................................................................................................................................................................  25     0   LCK-1
    ......................ggauggucgaaggagagagaug.............................................................................................................................................................................................................  25     0   LCK-2
    ................................aggaAagagaugggugga.......................................................................................................................................................................................................  1      1   JT-3
    .................................................................uuucugggucgaaggauaggaug.................................................................................................................................................................  2      0   LT-2
    ...................................................................ucugggucgaaggauagg....................................................................................................................................................................  1      0   LT-3
    ................................................................................................uucgagaauucuucAauga......................................................................................................................................  1      1   JT-1
    .......................................................................................................................................................................................................................aucggaucugagaugaga................  1      0   LT-1
```
